# Does board gender diversity weaken or strengthen executive risk-taking incentives?

**DOI:** 10.1371/journal.pone.0258163

**Published:** 2021-10-11

**Authors:** Pattanaporn Chatjuthamard, Pornsit Jiraporn, Sang Mook Lee

**Affiliations:** 1 Center of Excellence in Management Research for Corporate Governance and Behavioral Finance SASIN School of Management Chulalongkorn University Bangkok, Bangkok, Thailand; 2 Great Valley School of Graduate Professional Studies, The Pennsylvania State University, Malvern, PA, United States of America; The Bucharest University of Economic Studies, ROMANIA

## Abstract

We investigate the effect of board gender diversity on managerial risk-taking incentives. Our results demonstrate that companies with stronger board gender diversity provide more powerful executive risk-taking incentives. It appears that female directors’ risk aversion exacerbates managers’ risk aversion, resulting in a sub-optimal level of risk-taking. To offset this tendency for too little risk, companies are induced to provide stronger risk-taking incentives. Specifically, an increase in board gender diversity by one standard deviation raises vega by 10.3%. Further analysis corroborates the results, including propensity score matching, entropy balancing, and an instrumental-variable analysis. Endogeneity appears to be unlikely, suggesting that female directors are not merely associated with, but probably bring about stronger risk-taking incentives.

## Introduction

Several recent events have highlighted the importance of board gender diversity. For instance, the governor of California in 2018 signed a law requiring public companies whose principal executive offices were in California to have at least one female director on the board. Similarly, several European countries introduced gender quotas on corporate boards (for instance, Norway, Finland, France, Spain, Iceland, and Denmark). Therefore, the significance of board gender diversity could not be overemphasized. Not surprisingly, there is an immense literature on female executives and board gender diversity [1–6].

We enrich this crucial area of the literature by investigating the effect of board gender diversity on executive risk-taking incentives. It is well-known that managers exhibit risk aversion because, unlike diversified shareholders, they are more exposed to undiversifiable risk due to their firm-specific human capital [7–9]. Stock options are thus provided to induce managers to engage in more risk-taking. The importance of this branch of research is demonstrated by the existence of a large literature on managerial risk-taking incentives [9–16].

Following the literature in this area, we employ vega as our measure of risk-taking incentives. Vega is the change in the manager’s wealth in terms of dollars for a 1% change in the standard deviation of stock returns. A higher value of vega implies stronger risk-taking incentives [10, 17, 18]. Based on a large sample of almost 12,000 observations across nearly 20 years, our results demonstrate that higher board gender diversity leads to stronger managerial risk-taking incentives. More powerful incentives are required to counteract the risk aversion brought about by the presence of more female directors on the board. We control for firm fixed effects in our regression analysis. So, our results are unlikely driven by any time-invariant unobservable firm-specific characteristics.

Moreover, we execute several robustness checks to reduce endogeneity further. First, we perform propensity score matching (PSM), where an observation in the treatment group is carefully matched with an observation outside the treatment group that is most similar based on several firm and board characteristics [19, 20]. The PSM results confirm our conclusion. Second, we utilize entropy balancing, a novel matching technique to ensure that the distributions of the variables in the treatment and the control groups are comparable [21]. Furthermore, we run an instrumental-variable analysis using two alternate instruments. All the robustness checks show that more female directors result in more powerful executive risk-taking incentives. Endogeneity does not appear to be likely in our study. Our results are thus more likely to demonstrate causality, rather than merely an association.

Our results aptly enhance several vital areas of the literature. First, we contribute to the rich and large area of the literature on board gender diversity [1–4, 22–25]. While prior research has explored the effect of board gender diversity on various corporate outcomes, our study is the first to examine the effect of board gender diversity on managerial risk-taking incentives. We show that the proportion of female directors on the board has a palpable effect on how companies design their managerial risk-taking incentives.

Second, our study extends the literature on managerial risk-taking incentives as we demonstrate that board gender diversity is one of the significant determinants of risk-taking incentives [9–16]. Our results robustly support the arguments underlying the laws and regulations that promote gender diversity. Female directors are appointed not merely as tokens. Rather, they appear to exert a significant influence on important corporate policies and strategies, such as executive risk-taking incentives.

## Prior research and hypothesis development

### Managerial risk aversion

Managers frequently have the power to alter the extent of corporate risk-taking through the investment initiatives they choose. Managers are able to mitigate firm risk by selecting projects with lower cash flow volatility or investing in assets that help stabilize the firm’s income stream, such as diversification activities [9]. Numerous studies presume that when risk-averse managers are offered the choice, they are inclined to take on suboptimal corporate risk. They do so in order to safeguard their firm-specific human capital [7–9] and perquisite consumption [26], both of which are imperiled by firm risk [9]. In contrast to well-diversified shareholders, who prefer to accept all NPV projects irrespective of their project risk, managers may decline risk-increasing, positive NPV initiatives if the cost of the increased risk outweighs the benefit of improved firm value [9].

According to economic theory, stock options provide managers with incentives to overcome their risk aversion and pursue more riskier investments [7]. In support of this argument, prior research demonstrates that, when managers are offered stock options, they engage in more risky initiatives, bringing the level of corporate risk-taking closer to the optimal level [10, 17, 18].

### Board gender diversity and corporate governance

In the literature, several arguments have been made as to why board gender diversity should improve the quality of board governance. More board gender diversity brings about more diverse perspectives, which help correct informational biases in strategy formulation and problem solving [27–29]. Likewise, resource dependency theory establishes a theoretical framework for the board of directors’ role as a firm’s resource [30, 31]. Thus, it can be argued that female board members improve the firm’s governance by bringing different talents, competencies, and views to the table and by adding new dynamics to board deliberations [32]. According to resource dependency theory, board gender diversity is one of the crucial aspects of board governance and is expected to strengthen board effectiveness.

According to agency theory, the board’s role in an agency framework is to alleviate agency conflicts between managers and shareholders. One of the critical components of an agency perspective of the board is that outside directors would not collude with inside directors to undermine shareholder interests, as directors have incentives to establish reputations as expert monitors. Board independence is crucial for boards to act in shareholders’ best interests. It can be reasonably argued that diversity promotes board independence because members of a different gender, race, or cultural background may ask questions that directors from more typical backgrounds would not. Hence, board gender diversity should be beneficial and improve the functioning of the board [33].

### Board gender diversity and risk-taking

According to research both in psychology and economics, women are more risk averse than men. In experimental settings, males are more likely than women to make dangerous choices. In trials involving lotteries with known probability and monetary rewards, for example, women are found to be more risk averse [4, 34, 35]. Furthermore, women are more conservative when it comes to making investing decisions [4, 36, 44].

The studies above, however, focus on the differences in risk aversion between men and women in the general population. It is unclear if their conclusions can be extended to the boardroom. Arguably, women who make it to the boardroom are unlikely to share similar characteristics with those in the general population. Notwithstanding, given the fundamental differences in risk preferences between men and women, it is possible that board gender diversity may influence the extent to which companies are engaged in risk-taking [4].

The empirical evidence on the effect of board gender diversity on risk-taking is somewhat ambiguous. Many studies support the notion that higher board gender diversity leads to less risk-taking. For instance, according to Faccio, Marcicha, and Mura [5], European companies led by female CEOs exhibit lower leverage, less volatile earnings, and a greater likelihood of survival than those led by male CEOs. Additionally, transitioning from male to female CEOs (or vice versa) is associated with economically and statistically significant decreases (increases) in corporate risk taking. Khaw, Liao, Tripe, and Wongchoti [6], examining Chinese firms, find that companies where the board of directors are comprised of male directors only take significantly higher risk.

Furthermore, Harjoto, Laksmana, and Yang [37] investigate board diversity, including gender diversity, demonstrate that firms with more diverse boards are more risk averse, spending less on capital expenditures, R&D, and acquisitions, and exhibiting lower volatilities of stock returns and accounting returns than those with less diverse boards. Examining banks in OECD countries, Gulamhussen and Santa [38] find that female directors lead to a significant reduction in risk-taking. Lenard, Yu, York, and Wu [39], measuring firm risk by the variability in stock returns, find that female directors diminish firm risk. Using an international sample from 27 developing countries, Mohsni, Otchere, and Shahriar [40] find that board gender diversity reduces both operating and financial risk significantly.

Other research in this area, however, does not find a significant connection between board gender diversity and corporate risk-taking. For example, Sila, Gonzalez, and Hagendorff [4], relying on a dynamic model analysis, report no association between female directors and corporate risk-taking. Using a sample of French firms, Bruna, Dang, Scotto, and Ammari [41] do not find any significant effect of board gender diversity on risk-taking.

Finally, a few studies argue and show that female directors bring about more risk-taking. For instance, Berger, Kick, Schaek [42], investigating German banks, find that banks with more board gender diversity exhibit higher portfolio risk. Poletti-Hughes and Briano-Turrent [43] report that female directors lead to higher risk-taking in Latin American companies.

### The effect of board gender diversity on executive risk-taking incentives

Although prior research has investigated the effect of board gender diversity on corporate risk-taking, our study is the first to explore the effect of board gender diversity on managerial risk-taking incentives. Based on the literature, we propose two opposing hypotheses.

#### The weakened incentives hypothesis

First, it could be argued that more female directors on the board leads to less powerful managerial risk-taking incentives. It is well known in the literature that women exhibit stronger risk aversion than men [4, 34–36, 44]. Moreover, several prior studies show that board gender diversity is associated with less risk-taking [5, 6, 37–40].

As the proportion of female directors on the board rises, board decisions increasingly reflect the risk preferences of the female directors. Female directors prefer less risk-taking and are thus in favor of less powerful risk-taking incentives. This view predicts that higher board gender diversity weakens executive risk-taking incentives.

#### The strengthened incentives hypothesis

By contrast, an argument can be made that more female directors bring about more powerful risk-taking incentives. First, there may be a substitution effect. Because female directors are in favor of less risk-taking, their risk preferences exacerbate managerial risk aversion. When combined, managers and female directors’ preferences for less risk results in corporate risk-taking policies and strategies that are considerably sub-optimal. As a result, more powerful risk-taking incentives are needed to offset this tendency for too little risk. The more female directors on the board, the stronger executives risk-taking incentives need to be. In other words, a lack of board gender diversity substitutes for managerial risk-taking incentives. This view therefore predicts that board gender diversity strengthens risk-taking incentives.

Furthermore, managerial risk aversion can be viewed as an agency problem to the extent that managers avoid risk-taking to the point where shareholder value is not maximized. Theory suggests that board gender diversity improves board effectiveness, meaning that board gender diversity helps mitigate agency problems more effectively [27–32, 34]. As a result, more gender-diverse boards are in favor of stronger risk-taking incentives so as to reduce agency problems further than less gender-diverse boards. This hypothesis thus predicts stronger risk-taking incentives as a consequence of higher board gender diversity.

## Sample construction and data description

Our primary database is COMPUSTAT, which contains information both about firm characteristics and executive stock options in the United States. Consistent with previous research, we use options’ vega to measure executive risk-taking incentives [10, 13, 15, 17, 18, 45, 46]. Vega reflects the executive’s wealth’s sensitivity to stock return volatility. Specifically, vega is the change in the manager’s wealth in terms of dollars for a 1% change in the standard deviation of stock returns. Because the Execucomp database covers just the top-five executives, we utilize the average vega of the top management team. A higher value of vega indicates stronger managerial risk-taking incentives.

The Institutional Shareholder Services (ISS) provides data on the board of directors, including board gender diversity. Following the literature, our measure of board gender diversity is the percentage of female directors on the board. Our final sample consists of 12,431 firm-year observations spanning the years 1996 to 2014, resulting in an imbalanced panel data set of US companies.

Based on the literature in this area, we include a number of control variables that may influence vega [13, 15]. More specifically, we include board size (the number of directors), board independence (the percentage of independent directors), firm size (Ln of total assets), profitability (EBIT/total assets), leverage (total debt/total assets), investments (capital expenditures/total assets), intangible assets (R&D/total assets and advertising expense/total assets), cash holdings (cash holdings/total assets), asset tangibility (fixed assets/total assets), discretionary spending (SG&A expense/total assets), dividend payouts (total dividends/total assets), and delta (sensitivity of the executive’s wealth to stock returns). To account for possible variation over time, we include year fixed effects. Importantly, to reduce the omitted-variable bias, we include firm fixed effects, which control for any time-invariant characteristics that remain constant over time. Table 1 shows the descriptive statistics for all the variables. Notably, the average percentage of female directors (board gender diversity) is 10.307%, suggesting that only about 10% of the directors are females. The degree of board gender diversity is comparable to the statistics reported in prior research. S2 Table shows board gender diversity by sub-period. Notably, the percentage of female directors appears to be increasing over time. S1 Table describes the variable definitions.

**Table 1:** Summary statistics.

Variable	Mean	Std. Dev.	Median	25th	75th
Risk-taking Incentives					
Vega	60.042	69.801	32.773	8.911	87.773
Delta	411.776	1562.085	128.827	47.929	325.073
Board Characteristics					
Board gender diversity	10.307	9.594	10.000	0.000	16.667
% Independent Directors	73.272	15.618	76.923	66.667	85.714
Board Size	9.186	2.519	9.000	7.000	11.000
Average Director Tenure	9.049	13.382	8.429	6.222	11.000
Average Director Age	61.095	4.552	61.300	58.444	63.917
Board Gender Diversity (Comp.)	11.606	16.915	0.000	0.000	25.000
Board Independence (Comp.)	95.516	13.959	100.000	100.000	100.000
Firm Characteristics					
Ln (Total Assets)	7.651	1.612	7.512	6.475	8.651
EBIT/Total Assets	0.088	0.102	0.082	0.041	0.013
Total Debt/Total Assets	0.216	0.185	0.198	0.055	0.331
Capital Expenditures/Total Assets	0.050	0.057	0.034	0.015	0.065
Advertising Expense/Total Assets	0.011	0.034	0.000	0.000	0.004
R&D Expense/Total Assets	0.026	0.056	0.000	0.000	0.028
Dividends/Total Assets	0.013	0.029	0.004	0.000	0.017
Cash Holdings/Total Assets	0.143	0.163	0.078	0.025	0.206
Fixed Assets/Total Assets	0.503	0.436	0.392	0.157	0.787
SG&A Expense/Total Assets	0.193	0.202	0.142	0.026	0.290

Vega is sensitivity of the manager’s wealth to stock return volatility. Delta is the sensitivity of the manager’s wealth to stock returns. Board gender diversity is the percentage of female directors on the board. Board size is the number of directors on the board. SG&A is selling, administrative, and general expense.

## Results

### Baseline regression results

Table 2 shows the regression results where vega is the dependent variable. We include firm fixed effects to reduce any endogeneity bias arising from unobserved heterogeneity. Model 1 includes only the percentage of female directors on the board. The coefficient of board gender diversity is significantly positive. Model 2 includes all the control variables. Board gender diversity still carries a positive and significant coefficient. The results show that higher board gender diversity brings about stronger managerial risk-taking incentives, supporting the strengthened incentives hypothesis. To ensure that our results are robust to the estimation method, we run a random-effects regression in Model 3 and still obtain a similar result.

**Table 2:** The effect of board gender diversity on executive risk-taking incentives.

	(1)	(2)	(3)
	Firm-Fixed-Effects	Firm-Fixed-Effects	Random-Effects
	Vega	Vega	Vega
**Board Gender Diversity**	**1.271** ***	**0.349** ***	**0.334** ***
	**(14.357)**	**(3.769)**	**(2.746)**
Ln (Board Size)		10.671**	0.618
		(2.425)	(0.119)
% Independent Directors		-0.194***	-0.063
		(-3.588)	(-0.927)
Ln (Total Assets)		12.324***	21.967***
		(6.597)	(16.788)
EBIT/Total Assets		28.856***	40.980***
		(3.895)	(3.412)
Total Debt/Total Assets		-6.578	2.901
		(-1.193)	(0.417)
Capital Expenditures/Total Assets		30.845*	13.002
		(1.929)	(0.827)
Advertising Expense/Total Assets		62.363*	76.782**
		(1.696)	(2.339)
R&D Expense/Total Assets		19.964	100.607***
		(0.820)	(3.470)
Dividends/Total Assets		-25.656	-12.988
		(-1.274)	(-0.696)
Cash Holdings/Total Assets		2.988	19.075***
		(0.458)	(2.630)
Fixed Assets/Total Assets		-1.171	1.398
		(-0.286)	(0.379)
SG&A Expense/Total Assets		8.894	21.508***
		(1.029)	(2.831)
Delta		0.003***	0.003*
		(5.858)	(1.733)
Constant	46.589***	-119.529***	-130.865***
	(46.828)	(-7.248)	(-7.973)
Year Fixed Effects	Yes	Yes	Yes
Firm Fixed Effects	Yes	Yes	Yes
Observations	11,684	11,484	11,484
Adjusted R-squared	0.609	0.656	0.211

Vega is sensitivity of the manager’s wealth to stock return volatility. Delta is the sensitivity of the manager’s wealth to stock returns. Board gender diversity is the percentage of female directors on the board. Board size is the number of directors on the board. SG&A is selling, administrative, and general expense.

t-statistics in parentheses.

*** p<0.01

** p<0.05

* p<0.1.

We estimate the economic significance of the effect of board gender diversity as follows. One standard deviation of the percentage of female directors is 9.572. The coefficient of board gender diversity in Model 2 is 0.349. Therefore, an increase in board gender diversity by one standard deviation raises vega by 0.349 multiplied by 9.572, which is 3.341. Because the median value of vega is 32.429, a rise in board gender diversity by one standard deviation makes risk-taking incentives 10.301% more powerful (3.341 divided by 32.429). Consequently, the effect of female directors on managerial risk-taking incentives is not only statistically significant but is also economically meaningful.

### Propensity Score Matching (PSM)

Because we include firm fixed effects in our regressions, our results are unlikely to be affected by the endogeneity bias that can be attributed to unobserved heterogeneity. In any case, we perform further analysis to confirm that endogeneity is unlikely. To corroborate the results, we use propensity score matching [19, 20]. We specifically divide the sample into quartiles based on the percentage of female directors. Observations in the top quartile (greatest board gender diversity) are assigned to the treatment group. Then, for each observation in the treatment group, we select an observation in the remainder of the sample that is most comparable based on thirteen governance and firm characteristics (i.e. the thirteen control variables included in the regression analysis). We use the nearest neighbor method without replacement. So, with the exception of board gender diversity, our treatment and control firms are nearly identical across all observable parameters.

We run diagnostic testing to ensure that our matching is appropriate. Table 3 Panel A displays the results. Model 1 is a logistic regression with a binary dependent variable equal to one if the firm is in the treatment group (high board gender diversity) and zero otherwise. Model 1 covers the full sample (pre-match). The results show that the treatment firms differ considerably from the rest of the sample in many respects. Specifically, the treatment firms have more independent directors, have larger board size, are larger in size, make less capital investments, have more fixed assets, and have more discretionary spending. These material differences could complicate our analysis.

**Table 3:** Propensity Score Matching (PSM).

**Panel A: Diagnostic testing**			
	(1)		(2)
	Pre-Match		Post-Match
	Treatment		Treatment
% Independent Directors	0.037***		0.001
	(8.313)		(0.288)
Ln (Board Size)	0.700**		-0.434
	(2.480)		(-1.312)
Ln (Total Assets)	0.255***		0.001
	(5.463)		(0.018)
EBIT/Total Assets	-0.545		0.226
	(-0.979)		(0.370)
Total Debt/Total Assets	-0.089		0.006
	(-0.275)		(0.018)
Capital Expenditures/Total Assets	-5.333***		0.936
	(-3.818)		(0.533)
Advertising Expense/Total Assets	1.571		0.097
	(1.014)		(0.051)
R&D Expense/Total Assets	0.367		2.451
	(0.270)		(1.446)
Dividends/Total Assets	0.157		0.838
	(0.111)		(0.468)
Cash Holdings/Total Assets	0.556		-0.578
	(1.366)		(-1.283)
Fixed Assets/Total Assets	0.591***		-0.173
	(3.120)		(-0.705)
SG&A Expense/Total Assets	0.990***		-0.169
	(2.757)		(-0.416)
Delta	0.000		-0.000
	(0.144)		(-0.225)
Constant	-6.714***		0.007
	(-7.323)		(0.008)
Year Fixed Effects	Yes		Yes
Firm Fixed Effects	Yes		Yes
Pseudo R-squared	0.166		0.008
Observations	11,073		5,068
**Panel B: Regression analysis**			
		(1)	
		Vega	
**Board Gender Diversity**		**0.362** **	
		**(2.421)**	
Ln (Board Size)		26.294***	
		(3.127)	
% Independent Directors		-0.591***	
		(-4.792)	
Ln (Total Assets)		3.375	
		(0.864)	
EBIT/Total Assets		98.469***	
		(5.766)	
Total Debt/Total Assets		-0.861	
		(-0.075)	
Capital Expenditures/Total Assets		3.283	
		(0.081)	
Advertising Expense/Total Assets		16.196	
		(0.278)	
R&D Expense/Total Assets		61.797	
		(1.048)	
Dividends/Total Assets		14.696	
		(0.457)	
Cash Holdings/Total Assets		36.934***	
		(2.658)	
Fixed Assets/Total Assets		5.053	
		(0.480)	
SG&A Expense/Total Assets		-12.872	
		(-0.749)	
Delta		0.002***	
		(3.260)	
Constant		-82.645**	
		(-2.200)	
Year Fixed Effects		Yes	
Firm Fixed Effects		Yes	
Observations		5,068	
Adjusted R-squared		0.706	

Vega is sensitivity of the manager’s wealth to stock return volatility. Delta is the sensitivity of the manager’s wealth to stock returns. Board gender diversity is the percentage of female directors on the board. Board size is the number of directors on the board. SG&A is selling, administrative, and general expense.

Robust z-statistics in parentheses.

t-statistics in parentheses.

*** p<0.01

** p<0.05

* p<0.1.

Model 2 is a logistic regression for the propensity-score matched sample (post-match). In Model 2, none of the coefficients are significant. Therefore, our treatments and control firms are almost identical, statistically indistinguishable along all the observable dimensions. To the degree that board gender diversity is unimportant, our treatment and control firms should have indistinguishable vega. Table 3 Panel B shows the regression result using the propensity-score matched sample. The coefficient of board gender diversity remains significantly positive, once again corroborating the strengthened incentives hypothesis. Because our PSM results remain consistent, our conclusion does not appear to be principally driven by endogeneity.

### Entropy balancing

Previous research has been conducted on the stringent assumption of observable selection. To sidestep this assumption, we employ Hainmueller’s [21] entropy balancing methodology, an extension of conventional matching techniques. By adjusting for a vast variety of variables that may affect the treatment and control groups differently, entropy balancing corrects for self-selection caused by measurable characteristics. Entropy balancing, in particular, offers a high degree of covariate balance by utilizing a reweighting approach that directly includes covariate balance into the weight function applied to the sample units [21, 47]. Entropy balancing imposes a number of equilibrium constraints, requiring that the matching covariate distributions of the treatment and control groups in the preprocessed data match perfectly at all prespecified moments [21, 47]. Hainmueller [21] offers more detailed information regarding entropy balancing.

The following is how we execute entropy balancing. We identify firms whose board gender diversity is in the top quartile as our treatment group. The remainder of the sample is regarded as the control group. Then, we conduct entropy balancing on all of the control variables to ensure that the mean and the variance of the observations in the two groups are similar.

S3 Table shows the descriptive statistics for the variables before and after entropy balancing. Before entropy balancing, the means and the variances of the variables are different between the treatment and the control groups. After entropy balancing, however, the means and the variances of the variances are very close, suggesting that entropy balancing is successful. Table 4 displays the regression results for the entropy-balanced sample. The coefficient of board gender diversity is still positive and significant. More female directors bring about more powerful executive risk-taking incentives.

**Table 4:** Entropy balancing.

	(1)
	Vega
**Board Gender Diversity**	**0.214** **
	**(2.191)**
% Independent Directors	-0.415***
	(-5.485)
Ln (Board Size)	15.083***
	(2.966)
Ln (Total Assets)	6.738***
	(2.920)
EBIT/Total Assets	47.451***
	(4.921)
Total Debt/Total Assets	-0.017
	(-0.003)
Capital Expenditures/Total Assets	41.041*
	(1.692)
Advertising Expense/Total Assets	31.360
	(0.883)
R&D Expense/Total Assets	65.327*
	(1.766)
Dividends/Total Assets	-10.466
	(-0.513)
Cash Holdings/Total Assets	22.777***
	(2.691)
Fixed Assets/Total Assets	-8.932*
	(-1.693)
SG&A Expense/Total Assets	-1.213
	(-0.118)
Delta	0.002***
	(5.229)
Constant	-93.909***
	(-4.182)
Year Fixed Effects	Yes
Firm Fixed Effects	Yes
Observations	11,484
Adjusted R-squared	0.678

Vega is sensitivity of the manager’s wealth to stock return volatility. Delta is the sensitivity of the manager’s wealth to stock returns. Board gender diversity is the percentage of female directors on the board. Board size is the number of directors on the board. SG&A is selling, administrative, and general expense.

t-statistics in parentheses.

*** p<0.01

** p<0.05

* p<0.1.

### Instrumental-variable analysis

To confirm the robustness of our findings, we conduct an instrumental-variable analysis (IV). This technique mitigates the endogeneity biases caused by reverse causality, unobserved heterogeneity, and possible measurement errors. According to Knyazeva et al. [48], corporations prefer to hire directors locally. Firms located in close proximity share a local director pool and tend to have similar board composition. We build on Knyazeva et al. [48] finding and argue that companies located in the same geographic area should display similar board gender diversity due to the shared pool of potential female directors.

Our instrumental variable is the local pool of potential female directors i.e., the total number of female directors of all firms located in the same 3-digit zip code, excluding firm i. Companies located in an area with a larger supply of potential female directors should exhibit higher board gender diversity. In essence, we exploit the variation in the supply of potential female directors across the zip codes. Because zip codes are designed to maximize efficiency in mail delivery, they are not related to corporate outcomes or policies. Zip codes assignments are thus likely exogenous to firm characteristics. This technique based on zip code assignments has been adopted in the literature [49–51].

Table 5 shows the regression results. Model 1 is the first-stage regression where the dependent variable is the percentage of female directors. The coefficient of the total number of female directors of all firms in the same zip code is significantly positive, corroborating the findings in Knyazeva et al. [48] where companies tend to recruit directors locally. Model 2 is the second-stage regression where vega is the dependent variable. The coefficient of the percentage of female directors is positive and significant. So, the IV results confirm the strengthened incentives hypothesis as well, suggesting that our conclusion is robust to endogeneity.

**Table 5:** Instrumental-variable analysis using a geographic pool of female directors as instrument based on zip codes.

	(1)	(2)
	First Stage	Second Stage
	% Female Directors	Vega
**Local pool of Potential Female Directors (Zip Code)**	**0.059** ***	
	**(2.958)**	
**Board Gender Diversity (Instrumented)**		**6.234** *
		**(1.730)**
% Independent Directors	0.051***	-0.492**
	(8.368)	(-2.542)
Ln (Board Size)	1.476***	2.139
	(2.989)	(0.288)
Ln (Total Assets)	-0.206	13.570***
	(-0.982)	(5.745)
EBIT/Total Assets	-0.195	30.296***
	(-0.235)	(3.401)
Total Debt/Total Assets	-2.136***	5.874
	(-3.452)	(0.583)
Capital Expenditures/Total Assets	-3.365*	49.923**
	(-1.875)	(2.227)
Advertising Expense/Total Assets	-6.171	99.084**
	(-1.495)	(2.005)
R&D Expense/Total Assets	-0.890	25.816
	(-0.326)	(0.880)
Dividends/Total Assets	2.889	-42.759
	(1.278)	(-1.628)
Cash Holdings/Total Assets	0.899	-2.071
	(1.227)	(-0.246)
Fixed Assets/Total Assets	-0.036	-1.106
	(-0.078)	(-0.225)
SG&A Expense/Total Assets	0.287	6.344
	(0.295)	(0.607)
Delta	0.000	0.002***
	(0.695)	(4.330)
Constant	-6.108***	
	(-3.301)	
Year Fixed Effects	Yes	Yes
Firm Fixed Effects	Yes	Yes
Observations	11,484	11,128
Adjusted R-squared	0.771	0.726

Vega is sensitivity of the manager’s wealth to stock return volatility. Delta is the sensitivity of the manager’s wealth to stock returns. Board gender diversity is the percentage of female directors on the board. Board size is the number of directors on the board. SG&A is selling, administrative, and general expense. The local pool of potential female directors is the total number of female directors of all firms in the same geographical area defined by either a zip code or a city.

t-statistics in parentheses.

*** p<0.01

** p<0.05

* p<0.1.

In any event, just to be certain, we apply an alternate instrumental variable, which is the pool of potential female directors in the same city. This is calculated as the total number of female directors of all firms located in the same city, excluding firm i. The same logic applies here as earlier. However, our definition of an area is now different. The regression results are shown in Table 6. Model 1 and Model 2 are the first-stage and second-stage regressions respectively. The results remain similar. So, we continue to obtain consistent results after using two alternate instrumental variables. Our conclusion is quite robust.

**Table 6:** Instrumental-variable analysis using a geographic pool of female directors as instrument based on cities.

	(1)	(2)
	First Stage	Second Stage
	% Female Directors	Vega
**Local pool of Potential Female Directors (City)**	**0.070** ***	
	**(3.916)**	
**Board Gender Diversity (Instrumented)**		**5.867** **
		**(2.195)**
% Independent Directors	0.051***	-0.473***
	(8.366)	(-3.168)
Ln (Board Size)	1.515***	2.671
	(3.068)	(0.414)
Ln (Total Assets)	-0.197	13.492***
	(-0.939)	(5.955)
EBIT/Total Assets	-0.143	30.206***
	(-0.171)	(3.462)
Total Debt/Total Assets	-2.156***	5.097
	(-3.485)	(0.593)
Capital Expenditures/Total Assets	-3.452*	48.732**
	(-1.924)	(2.357)
Advertising Expense/Total Assets	-6.116	96.792**
	(-1.482)	(2.091)
R&D Expense/Total Assets	-0.761	25.451
	(-0.279)	(0.886)
Dividends/Total Assets	3.027	-41.692*
	(1.340)	(-1.675)
Cash Holdings/Total Assets	0.844	-1.755
	(1.153)	(-0.219)
Fixed Assets/Total Assets	-0.037	-1.110
	(-0.079)	(-0.231)
SG&A Expense/Total Assets	0.229	6.503
	(0.236)	(0.637)
Delta	0.000	0.002***
	(0.674)	(4.499)
Constant	-6.212***	
	(-3.358)	
Year Fixed Effects	Yes	Yes
Firm Fixed Effects	Yes	Yes
Observations	11,484	11,128
Adjusted R-squared	0.771	0.723

Vega is sensitivity of the manager’s wealth to stock return volatility. Delta is the sensitivity of the manager’s wealth to stock returns. Board gender diversity is the percentage of female directors on the board. Board size is the number of directors on the board. SG&A is selling, administrative, and general expense. The local pool of potential female directors is the total number of female directors of all firms in the same geographical area defined by either a zip code or a city.

t-statistics in parentheses.

*** p<0.01

** p<0.05

* p<0.1.

#### The role of board gender diversity on the compensation committee

It could be argued that the effect of board gender diversity on executive risk-taking incentives may go through the compensation committee, rather than the entire board of directors [52]. The compensation committee plays a crucial role in setting executive compensation schemes including managerial risk-taking incentives. To explore this possibility, we include two additional variables from the compensation committee, namely the percentage of female directors on the compensation committee and the percentage of independent directors on the compensation committee.

In addition, to demonstrate that our results are robust to additional control variables, we add a few board attributes as control variables. In particular, we add the CEO pay slice, which has been widely used in the literature as a proxy for CEO power [53]. We also add average director age and average director tenure as director tenure and director age have been found to be important attributes of the board of directors in the literature [54, 55].

The regression results are shown in Table 7. In Model 1, we include the percentage of female directors on the compensation committee (board gender diversity on the compensation committee) without including the percentage of female directors on the full board. The coefficient of board gender diversity on the compensation committee is not statistically significant. Therefore, it does not appear that executive risk-taking incentives are influenced by board gender diversity on the compensation committee. In Model 2, we add board gender diversity for the whole board as an independent variable. The coefficient of board gender diversity for the full board is positive and significant, confirming the results reported earlier. In conclusion, there is no evidence that board gender diversity on the compensation committee is one of the critical determinants of executive risk-taking incentives. Rather, it is board gender diversity of the full board that matters.

**Table 7:** The role of board gender diversity on the compensation committee.

	(1)	(2)
	vega	vega
Board Gender Diversity		0.236**
		(2.090)
Board Gender Diversity on Compensation Committee	-0.031	-0.071
	(-0.741)	(-1.534)
% Independent Directors on Compensation Committee	-0.000	0.005
	(-0.000)	(0.101)
CEO Pay Slice	24.813***	24.779***
	(4.475)	(4.470)
Average Director Age	-0.329*	-0.292
	(-1.829)	(-1.616)
Average Director Tenure	0.007	0.010
	(0.228)	(0.308)
% Independent Directors	-0.200***	-0.213***
	(-2.835)	(-3.013)
Ln (Board Size)	12.624**	12.144**
	(2.517)	(2.419)
Ln (Total Assets)	14.221***	14.236***
	(6.676)	(6.685)
EBIT/Total Assets	31.179***	31.129***
	(3.736)	(3.731)
Total Debt/Total Assets	0.472	0.850
	(0.077)	(0.138)
Capital Expenditures/Total Assets	35.071*	35.966*
	(1.907)	(1.956)
Advertising Expense/Total Assets	48.885	52.183
	(1.124)	(1.199)
R&D Expense/Total Assets	7.868	8.641
	(0.282)	(0.309)
Dividends/Total Assets	-25.047	-25.582
	(-1.157)	(-1.182)
Cash Holdings/Total Assets	4.023	3.889
	(0.554)	(0.536)
Fixed Assets/Total Assets	1.454	1.424
	(0.319)	(0.312)
SG&A Expense/Total Assets	27.255***	27.021***
	(2.640)	(2.617)
Delta	0.002***	0.002***
	(4.496)	(4.492)
Constant	-116.528***	-118.444***
	(-5.495)	(-5.581)
Firm Fixed Effects	Yes	Yes
Year Fixed Effects	Yes	Yes
Observations	9,630	9,630
Adjusted R-squared	0.672	0.672

t-statistics in parentheses.

*** p<0.01

** p<0.05

* p<0.1.

## Conclusions

Board gender diversity has attracted a great deal of attention lately due to several of the recent developments that promote gender diversity. Several European countries have gone as far as imposing board gender quotas on their public firms. Similarly, the debate over the costs and benefits of board gender diversity in the academic literature continues unabated. We contribute to the literature by exploring the influence of female directors on executive risk-taking incentives. Our results demonstrate that more gender-diverse boards result in more powerful managerial risk-taking incentives. The findings are consistent with the notion that female directors’ risk aversion, combined with managers’ risk aversion, results in a sub-optimal degree of risk-taking. To offset this bias for too little risk, companies provide more powerful risk-taking incentives. In particular, when the percentage of female directors rises by one standard deviation, vega increases by 10.3%, an economically meaningful magnitude.

Additional analysis corroborates the results, including fixed- and random- effects regressions, propensity score matching (PSM), entropy balancing, and an instrumental-variable analysis. Our results survive all the robustness checks and thus do not appear to be driven by endogeneity. Consequently, our results probably demonstrate causality, rather than merely an association. We combine two crucial branches of the literature i.e., board gender diversity and managerial risk-taking, and demonstrate that female directors are not merely tokens. They exert a palpable influence on important corporate policies, such as executive risk-taking incentives.

The results of our study have a few policy implications. For instance, several countries have imposed or plan to impose a mandate on board gender diversity. While some believe that such regulations may not lead to any concrete outcomes because women may be merely tokens on the board, our results demonstrate that board gender diversity exerts a significant influence on a crucial corporate outcome, i.e. executive risk-taking incentives. Therefore, regulators should exercise caution when imposing any requirements on board gender diversity as it does matter a great deal. Furthermore, investors and regulators that pay attention to the extent of corporate risk-taking should be informed by our results. Board governance, such as board gender diversity, is one of the crucial determinants of managerial risk-taking incentives. So, any attempt to understand the nature of corporate risk-taking should take into account the gender diversity on the board.

## Supporting information

S1 Table Variable definitions. (DOCX)

S2 Table Board gender diversity by sample period. (DOCX)

S3 Table Summary statistics before and after entropy balancing. (DOCX)
