# Supplementary material for: Does board gender diversity weaken or strengthen executive risk-taking incentives?
Source: PLoS One. 2021 Oct 11;16(10):e0258163. doi: 10.1371/journal.pone.0258163 (PMC8504771; doi:10.1371/journal.pone.0258163)
Supplement: S1 Table — (DOCX) [file pone.0258163.s001.docx]

**Table A1: Variable definitions**

| Variable | Definition |  |  |
| --- | --- | --- | --- |
| Risk-taking Incentives |  |  |  |
| Vega | Change in the manager's wealth in terms of dollars for a 1% change in the standard deviation of stock returns. | | |
| Delta | Change in the manager's wealth in terms of dollars for a 1% change in stock returns. | | |
| Board Characteristics |  |  |  |
| Board Gender Diversity | Percentage of Female Directors | | |
| Board Independence | Percentage of Independent Directors | | |
| Board Size | Number of Directors on the board | | |
| CEO Power | CEO Pay Slice (Bebchuk, Cremers, and Peyers, 2011) | | |
| Director Tenure | Average Director Tenure | | |
| Director Age | Average Director Age | | |
| Board Gender Diversity (Comp) | Percentage of Female Directors on the Compensation Committee |  |  |
| Board Independence (Comp) | Percentage of Independent Directors on the Compensation Committee |  |  |
| Firm Characteristics |  |  |  |
| Firm Size | Ln (Total Assets) | |  |
| Profitability | EBIT/Total Assets | |  |
| Leverage | Total Debt/Total Assets | | |
| Capital Investments | Capital Expenditures/Total Assets | | |
| Advertising Expense | Advertising Expense/Total Assets | | |
| R&D Investments | R&D Expense/Total Assets | | |
| Dividend Payouts | Dividends/Total Assets | | |
| Cash Holdings | Cash Holdings/Total Assets | | |
| Asset Tangibility | Fixed Assets/Total Assets | | |
| Discretionary Spending | SG&A Expense/Total Assets |  |  |
