# Supplementary material for: Does board gender diversity weaken or strengthen executive risk-taking incentives?
Source: PLoS One. 2021 Oct 11;16(10):e0258163. doi: 10.1371/journal.pone.0258163 (PMC8504771; doi:10.1371/journal.pone.0258163)
Supplement: S2 Table — (DOCX) [file pone.0258163.s002.docx]

**Table A2: Board gender diversity by sample period**

The sample period is divided into four sub-periods. This table displays the summary statistics for board gender diversity for each period.

| Sub-Period | Mean | S.D. | Median | 0.25 | 0.75 |
| --- | --- | --- | --- | --- | --- |
|  |  |  |  |  |  |
| 1996-2002 | 6.543 | 8.022 | 0.000 | 0.000 | 11.111 |
| 2003-2007 | 9.589 | 9.081 | 10.000 | 0.000 | 15.385 |
| 2008-2010 | 11.367 | 9.595 | 11.111 | 0.000 | 18.182 |
| 2011-2014 | 12.893 | 10.057 | 12.500 | 0.000 | 20.000 |
