# Supplementary material for: Does board gender diversity weaken or strengthen executive risk-taking incentives?
Source: PLoS One. 2021 Oct 11;16(10):e0258163. doi: 10.1371/journal.pone.0258163 (PMC8504771; doi:10.1371/journal.pone.0258163)
Supplement: S3 Table — (DOCX) [file pone.0258163.s003.docx]

**Table A3: Summary statistics before and after entropy balancing**

**Panel A: Summary statistics for the treatment and the control groups before entropy balancing**

|  | Treatment | | Control | |
| --- | --- | --- | --- | --- |
|  | Mean | Variance | Mean | Variance |
|  |  |  |  |  |
| % Independent Directors | 80.050 | 136.500 | 71.340 | 257.700 |
| Ln (Board Size) | 2.371 | 0.038 | 2.269 | 0.062 |
| Ln (Total Assets) | 8.302 | 2.721 | 7.466 | 2.411 |
| EBIT/Total Assets | 0.091 | 0.007 | 0.087 | 0.011 |
| Total Debt/Total Assets | 0.236 | 0.030 | 0.211 | 0.035 |
| Capital Expenditures/Total Assets | 0.042 | 0.002 | 0.052 | 0.004 |
| Advertising Expense/Total Assets | 0.014 | 0.002 | 0.010 | 0.001 |
| R&D Expense/Total Assets | 0.018 | 0.002 | 0.028 | 0.003 |
| Dividends/Total Assets | 0.015 | 0.001 | 0.012 | 0.001 |
| Cash Holdings/Total Assets | 0.127 | 0.021 | 0.148 | 0.028 |
| Fixed Assets/Total Assets | 0.511 | 0.170 | 0.500 | 0.196 |
| SG&A Expense/Total Assets | 0.197 | 0.049 | 0.192 | 0.038 |
| Delta | 425.900 | 6465620.000 | 407.700 | 1293960.000 |

**Panel B: Summary statistics for the treatment and the control groups after entropy balancing**

|  | Treatment | | Control | |
| --- | --- | --- | --- | --- |
|  | Mean | Variance | Mean | Variance |
|  |  |  |  |  |
| % Independent Directors | 80.050 | 136.500 | 80.040 | 136.700 |
| Ln (Board Size) | 2.371 | 0.038 | 2.371 | 0.039 |
| Ln (Total Assets) | 8.302 | 2.721 | 8.302 | 2.721 |
| EBIT/Total Assets | 0.091 | 0.007 | 0.090 | 0.007 |
| Total Debt/Total Assets | 0.236 | 0.030 | 0.236 | 0.030 |
| Capital Expenditures/Total Assets | 0.042 | 0.002 | 0.042 | 0.002 |
| Advertising Expense/Total Assets | 0.014 | 0.002 | 0.014 | 0.002 |
| R&D Expense/Total Assets | 0.018 | 0.002 | 0.018 | 0.002 |
| Dividends/Total Assets | 0.015 | 0.001 | 0.015 | 0.001 |
| Cash Holdings/Total Assets | 0.127 | 0.021 | 0.127 | 0.021 |
| Fixed Assets/Total Assets | 0.511 | 0.170 | 0.511 | 0.170 |
| SG&A Expense/Total Assets | 0.197 | 0.049 | 0.197 | 0.050 |
| Delta | 425.900 | 6465620.000 | 425.900 | 6463154.000 |
|  |  |  |  |  |
